# Hard X-ray imaging and tomography at the Biomedical Imaging and Therapy beamlines of Canadian Light Source

**DOI:** 10.1107/S1600577524005241

**Published:** 2024-07-15

**Authors:** Sergey Gasilov, M. Adam Webb, Arash Panahifar, Ning Zhu, Omar Marinos, Toby Bond, David M. L. Cooper, Dean Chapman

**Affiliations:** a Canadian Light Source 44 Innovation Boulevard SaskatoonS7N 2V3 Canada; b College of Medicine University of Saskatchewan 107 Wiggins Road SaskatoonS7N 5E5 Canada; Bhabha Atomic Research Centre, India

**Keywords:** hard X-rays, microtomography, *in vivo* imaging, time-resolved studies

## Abstract

The Biomedical Imaging and Therapy facility of the Canadian Light Source comprises two beamlines, which together cover a wide X-ray energy range from 13 keV up to 140 keV. The beamlines were designed with a focus on synchrotron applications in preclinical imaging and veterinary science as well as microbeam radiation therapy. While these remain a major part of the activities of both beamlines, a number of recent upgrades have enhanced the versatility and performance of the beamlines, particularly for high-resolution microtomography experiments. As a result, the user community has been quickly expanding to include researchers in advanced materials, batteries, fuel cells, agriculture, and environmental studies. This article summarizes the beam properties, describes the endstations together with the detector pool, and presents several application cases of the various X-ray imaging techniques available to users.

## Abstract 2

The current state of the Biomedical Imaging and Therapy beamlines of the Canadian Light Source is described and new capabilities are presented.

## 1. Introduction

The Canadian Light Source (CLS) is Canada’s national synchrotron facility and is located in the grounds of the University of Saskatchewan in the city of Saskatoon. CLS has a third-generation 2.9 GeV storage ring which presently operates in top-up mode with 220 mA ring current. CLS was officially opened in 2005 and currently has 22 operational beamlines. The two BioMedical Imaging and Therapy (BMIT) beamlines were built during the second phase of construction with the first experiments on the BMIT bending magnet beamline (BMIT-BM; Wysokinski *et al.*, 2007 ▸) taking place in 2010, while construction of the BMIT wiggler-based beamline (BMIT-ID; Wysokinski *et al.*, 2015 ▸) was completed by 2015. BMIT was designed to primarily serve a growing community of biomedical scientists interested in multidisciplinary approaches to the study of biological processes and diseases. Since this involves working with various animal models, much attention has been given to the imaging of large, heavy samples at medium and low spatial resolutions. The BMIT-ID monochromator has been optimized for a high-energy beam with a very large cross-section (Wysokinski *et al.*, 2018 ▸). Instruments for vertical expansion of the beam have also been proposed (Martinson *et al.*, 2014 ▸). BMIT-ID is certainly unique in North America as no other beamline can deliver a 100 keV monochromatic X-ray beam with the usable size of 110 mm × 6 mm (horizontal and vertical directions, respectively). X-ray tomography of such a large animal as a dog had been attempted *in vivo* with 54 µm voxel size (Cardoso, 2019 ▸).

While development of live-animal imaging on BMIT has been ongoing, researchers in other areas have also expressed significant interest in X-ray microtomography (µCT). In order to meet the strong demand for high-resolution imaging, µCT endstations were deployed on both the BMIT-BM and BMIT-ID beamlines. The detector pool was upgraded, and new data acquisition and data reconstruction systems were introduced in 2019–2021. In addition to outstanding biomedical research (Harrison *et al.*, 2020 ▸; Cooper *et al.*, 2023 ▸; Helpard *et al.*, 2020 ▸; Luan *et al.*, 2017 ▸), BMIT instruments were also applied to the design of advanced materials (Capilli *et al.*, 2022 ▸), understanding of failure mechanism in batteries (Bond *et al.*, 2022 ▸), agriculture and food chemistry (Chen *et al.*, 2021 ▸), paleontology (Brinkman *et al.*, 2023 ▸), environmental studies (Leontowich *et al.*, 2022 ▸), and other disciplines.

In this paper we describe the BMIT sources, beamline layouts, and beam properties as well as introduce the current state of user endstations, the detector pool, and software/hardware for data acquisition and reconstruction. We then show several research highlights and discuss existing limitations together with the present upgrade plans.

## 2. Overview, source and beam properties

**Figure 1:**
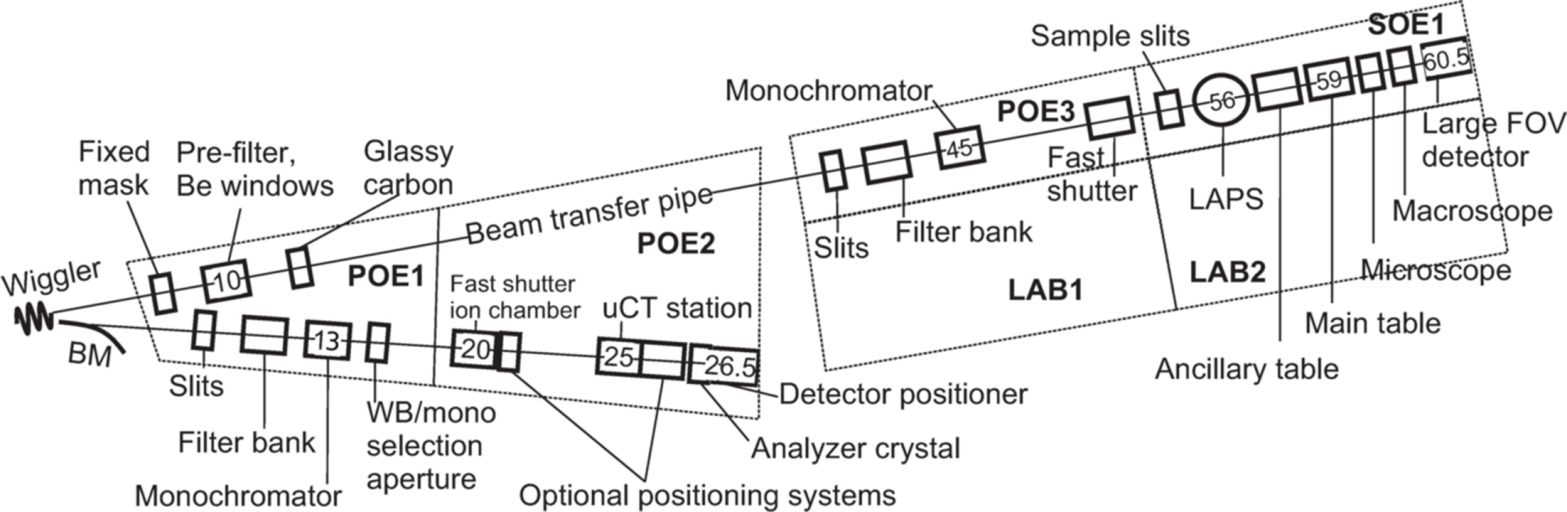
Schematic layout of the BMIT beamlines. Distances from the source to several selected components are given (in metres) for the sense of scale. LAB1 and LAB2 are laboratories adjacent to the BM control room and SOE1 hutches. LAPS stands for large animal position system (see Section 3.2).

The layout of the BMIT facility is shown schematically in Fig. 1 ▸. BMIT has two experimental hutches which can be used simultaneously. The bending magnet beam (BMIT-BM) is delivered to the POE2 hutch where it is used for experiments on smaller, lightweight samples while the high-energy beam from the wiggler (BMIT-ID) is used to penetrate dense materials and image large samples in SOE1 (POE and SOE stand for primary and secondary optical enclosures, respectively). The sections below enumerate the key parameters of these two sources and describe beam conditioning optics and the resulting beam properties.

### 2.1. BMIT-BM: lower energy bending magnet beamline

**Figure 2:**
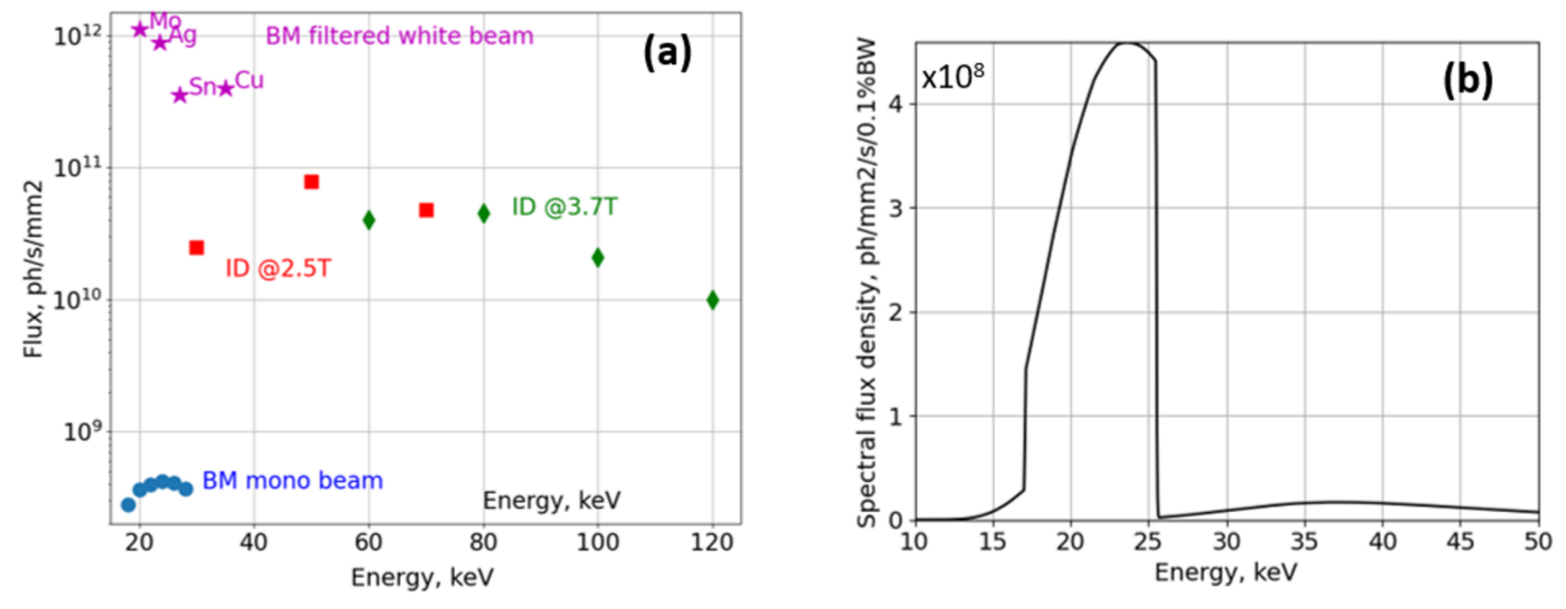
(*a*) Comparison of typical photon flux density in white and monochromatic beams at the sample position. Values are measured with an ionization chamber and a radiometer for the BMIT-BM and BMIT-ID beamlines, respectively; measurements in the 15–60 keV range are in a very good agreement with those made with a diode calibrated at BAM*line* (BESSY II, Germany). Star-shaped markers show integrated flux in the filtered white beam. The letters next to each star-shaped marker indicate the material of the filter used to shape the radiation spectrum and each star is positioned at the weighted mean energy of the resulting polychromatic beam. Panel (*b*) shows the effective spectrum of the BMIT-BM beam filtered by a 0.1 mm-thick Ag filter and registered by a 20 µm-thick LuAG scintillator [computed with the help of the *XRT* software package (Klementiev & Chernikov, 2014 ▸)]. Note that for such a thin scintillator more than 95% of the detected photons are confined in the ∼17–25 keV spectral range and the flux density is about 2 × 10^11^ photons mm^−2^ s^−1^.

BMIT-BM is a full-field X-ray imaging beamline based on a bending magnet source. The layout and dimensions are similar to µCT beamlines built at other 2.5–3 GeV rings in 2000–2010 (Rack *et al.*, 2008 ▸, 2009 ▸; Stampanoni *et al.*, 2007 ▸). The source and beam properties are listed in Table 1 ▸. X-ray optics are simple: a set of filters and an optional monochromator. The monochromator is located 13 m from the source and operates in Bragg geometry with two very large Si [2,2,0] crystals providing monochromatic beam in the 15–40 keV energy range. Its bandwidth is too small for projects which require µCT imaging with voxel size of less than 5 µm. On the other hand, owing to the exceptional stability of the crystals, the monochromator is perfect for analyzer-crystal-based radiographic imaging with a pixel size of 20 µm (Aulakh *et al.*, 2017 ▸). The ultra-small-angle scattering contrast enabled by rocking curve imaging (Wernick *et al.*, 2003 ▸) is used by researchers who study lung diseases and test the effects of, for instance, anti-fibrotic drugs. For µCT with a voxel size of less than 5 µm (down to 0.36 µm) the monochromator crystals are moved out of the beam path and the filtered white beam is used. The spectral flux is shaped by (1) attenuators such as glassy carbon, Al, and Cu, and (2) thin Mo, Ag, and Sn plates whose *K*-edges help to preferentially attenuate higher-energy photons and set the mean beam energy to ∼20, 23 and 26 keV, respectively. In combination with thin scintillator crystals, the fraction of high-energy X-rays contributing to images is effectively around 2–5% [Fig. 2 ▸(*b*)].

### 2.2. BMIT-ID: high energy wiggler beamline

BMIT-ID was conceived as a beamline for low-resolution hard X-ray imaging of large samples and animals, such as dogs and sheep, as well as for microbeam radiation therapy research. The source is a superconducting wiggler with 26 full-field periods of 48 mm period length procured from the Budker Institute for Nuclear Physics (Wysokinski *et al.*, 2016 ▸). The original conceptual design of BMIT-ID resembles the ID17 beamline at ESRF: either an extremely intense white beam is applied for irradiation in the POE2 hutch or monochromatic beam (Table 2 ▸) is used for experiments in SOE1 (see Fig. 1 ▸). In the end, from practical and performance standpoints, BMIT-ID is more comparable with the JEEP beamline (Drakopoulos *et al.*, 2015 ▸) of the Diamond Light Source, as both CLS and Diamond storage rings operate at ∼3 GeV energy. In order to generate a significant amount of hard X-rays, a superconducting wiggler with high *K*-value and many poles is required. On a 3 GeV ring such a device produces a lot of power in X-rays below the critical energy. Depending on the magnetic field the critical energy varies from 14 keV at 2.5 T up to 21 keV at 3.8 T (for comparison, the ESRF ID17 wiggler operating at a magnetic field of 1.4 T achieves the critical energy around 30 keV). At the latter magnetic field, the BMIT wiggler generates 21 kW of power and with the angular acceptance of the front-end apertures set to 4.0 mrad × 0.3 mrad the X-ray beam carries 13 kW of power down the beamline. Combined with very short distances to the first optical components, the absorbed power densities in the front-end window and the POE1 attenuators can be as high as 20 W mm^−2^. This creates a nearly insurmountable problem of heat damage to virtually any material if it is closer than 40 m to the source. Only a diamond-based filter can withstand the power densities occurring in the POE1 hutch; however, it cannot dissipate enough heat. Therefore, the diamond filter is complemented by SiC absorbers located at ∼42 m from the source and only after that a monochromator can be used.

The BMIT-ID monochromator is based on a bent Laue, double-crystal, fixed-exit design (Schulze *et al.*, 1994 ▸) and it is the key component of the beamline. It is equipped with two 20 cm-wide, 2 mm-thick Si[111] crystals and a bending mechanism; the accessible spectral range is from 26 keV to 140 keV (Wysokinski *et al.*, 2018 ▸). The monochromator is water-cooled by a gravity-fed system in order to minimize vibrations. The first crystal is located at ∼44.5 m from the source. The upstream beamline slits are typically opened to 180 mm × 10 mm to expose an area larger than the full width at half-maximum (FWHM) of the beam intensity footprint and mitigate the formation of the heat bump. With such settings, the unfiltered wiggler beam would deposit 6 kW of power in the crystal and the peak power density would exceed 3 W mm^−2^. The first crystal of the monochromator would not withstand such a heat load. Finite-element analysis has been performed taking into account both mechanical stress due to bending and thermal load of the wiggler beam. The results of this numerical modeling showed that, in order to operate with stresses below 50% of the critical stress in Si, the absorbed power in the crystal must not exceed 600 W and the peak absorbed power density should be less than 0.5 W mm^−2^. In practice, soon after the absorbed power exceeds ∼500 W, the crystal cannot achieve a thermal equilibrium which manifests itself in a very poor pointing stability of the exiting monochromatic beam. These instabilities give rise to a rather non-uniform intensity distribution, which changes over time and results in severe ring artifacts in the tomographic reconstructions.

### 2.3. Heat-load mitigation and attainable photon flux densities

To reduce the heat load to acceptable levels, about 11.5 kW of power must be taken away from the beam before it interacts with the first crystal. Even though BMIT-ID is the longest CLS beamline, any water-cooled metal plate will be inevitably damaged if exposed to such power densities. To circumvent the problem while retaining a decent filter transmission at 30 keV, the beam is attenuated in multiple stages by a set of thin plates with materials of progressively increasing atomic number. Permanent pre-filters include a 0.5 mm-thick CVD diamond absorber, two 0.5 mm-thick vacuum-tight Be windows and 2 mm of graphite; all located at the distance of 10–15 m from the source. The bulk of the power is dissipated in a 2 mm-thick SiC filter located approximately 40 m from the source. This 2 mm-thick SiC plate purchased from Morgan Advance Materials (USA) can withstand 6 kW of heat with the peak density of 4 W mm^−2^. Additional SiC, Al, and Cu filters can be used depending on the selected wiggler field to maximize photon flux at the required photon energy while keeping the power absorbed in the monochromator crystal below 500 W.

**Figure 3:**
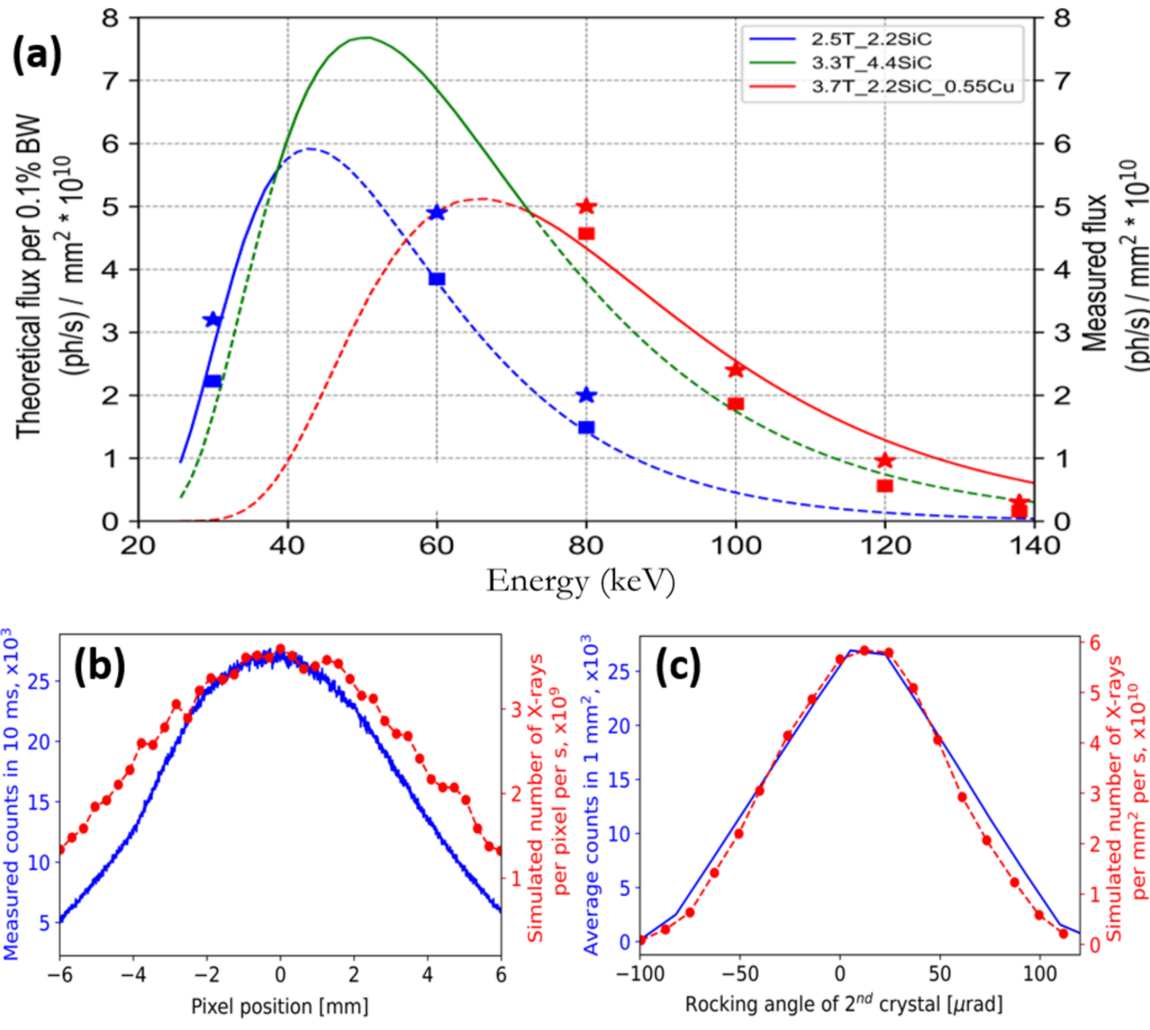
Properties of the BMIT-ID beam. Shown in (*a*) is the photon flux density through the centered 1 mm × 1 mm aperture at a distance of 58 m from the source: curves show spectral flux density (computed in *SPECTRA10*) of the beam attenuated by the mandatory filters and 4.4 mm of Si (combined thickness of the two monochromator crystals); flux densities at 2.5 T and 3.7 T and with the same sets of attenuators were measured with the radiometer and computed in *XRT* and are shown by squares and stars, respectively. Panel (*b*) shows the vertical profile of the monochromatic beam while panel (*c*) presents the rocking curve of the second monochromator crystal. In both cases the X-ray energy was 50 keV and a detector with 8 µm × 8 µm pixels was used to acquire the data; in both (*b*) and (*c*) blue solid lines are experimental values while dotted red lines were simulated in *XRT* (see text for details).

Typical photon flux density at the sample position is shown in Fig. 3 ▸(*a*) for the entire BMIT-ID operation range. Curves are photon fluxes calculated in *SPECTRA10* (Tanaka & Kitamura, 2001 ▸) in units of photons (0.1% bandwidth)^−1^ s^−1^ through a centered 1 mm × 1 mm aperture at a distance of 58 m from the source for a beam attenuated by the standard filters (listed in the legend) and by 4.4 mm of Si which is the thickness of two Si crystals of the double Laue monochromator that the beam must go through. Square markers show the number of photons through the 1 mm × 1 mm aperture measured with a liquid-He-cooled radiometer (developed at CLS, to be described in a separate publication) when the monochromator is tuned to the maximum flux. Measurements were performed at 2.5 T at 30, 60, and 80 keV and at 3.7 T at 80, 100, 120, and 140 keV. It was found that the relative flux in the higher harmonics is about 5% when working in the range 30–50 keV at 2.5 T wiggler field. This contribution from high harmonics increases to 30% if the wiggler field is 3.7 T. In practice, high wiggler fields are only used for experiments at energies above 50 keV when harmonics above 150 keV ([333], [444] and other high-order reflections from Si) can be effectively neglected again.

The shape of the monochromator crystals and the bending mechanism are described in detail by Wysokinski *et al.* (2018 ▸) but attainable flux densities are not reported in that work. A simplified idea of the bending mechanism is to induce a bending moment in the Si crystal by pushing on the free end of a steel strip whose other end is firmly attached to the corner of the Si crystal. We studied thoroughly the bending system as it determines the achievable flux gains compared with the flat crystals which is crucially important for the microtomography experiments. A very surprising finding was that the crystal is not being bent to a cylindrical shape. This observation was confirmed by a careful inspection of surface profiles obtained by means of finite-element analysis. Nonetheless, the lattice strain and surface deformations induced by bending are sufficient to boost intensity of the diffracted beam by a factor of 13 compared with the flat crystal. Further bending does not produce substantial gains. This happens for two reasons. Firstly, if the steel strips are pushed by more than 10 mm, this will result in plastic deformations and rapidly diminishing transfer of the force (note that if the strips are too thick they will break the crystal and if they are too thin they will not produce enough bending momentum even if their ends are pushed past 20 mm). Secondly, both displacement sensors and attempts to measure the curvature by means of focusing of the visible light showed that when we push the benders the crystal’s surface tilts as a whole in addition to being curved. Inspection of the finite-element analysis results indicates that this is related to the shape of the crystal itself and the pivot point which is located at the bottom of the 2 mm thin active region where this thin wall connects to the bulky crystal base.

We then found that upon diffraction in the second crystal almost half of the intensity is being lost. This is in good agreement with the simulations done in the *XRT* ray-tracing tool which has been recently updated and can now model Laue diffraction in thick, bent crystals (Klementiev & Chernikov, 2023 ▸). We used it to compute the flux density in the monochromatic beam [Fig. 3 ▸(*a*)], vertical profile of the beam intensity distribution [Fig. 3 ▸(*b*)], and the rocking curve of the second crystal [Fig. 3 ▸(*c*)]. A good agreement between the experiment and simulation has been obtained under the assumption that our first crystal is bent to a radius of 15 m and the second is bent to 20 m. The discrepancies in the computed [shown by star-shaped markers in Fig. 3 ▸(*a*)] and measured values of flux (squares) and the noticeable difference between the FWHM of the beam profiles are attributed to the aforementioned fact that the crystals are not being bent to a perfect cylindrical shape. Regarding the beam spatial profile, in practice we have a sufficiently uniform distribution of intensity across up to 120 mm × 8 mm of the beam footprint at 50 keV. The useful footprint decreases to 110 mm × 6 mm at 100 keV. Working with a larger beam is possible but intensity will drop by more than 50% at the image corners compared with the center which will result in a poor signal to noise ratio.

Interestingly, both the shape and FWHM of the rocking curve taken with the second crystal through a 1 mm × 1 mm aperture centered on the beam main axis are in outstanding agreement with the simulation. The energy spread along the vertical direction in the full beam (10 mm) was estimated using a Ba *K*-edge and was found to be just under 200 eV. This is also in a good agreement with the number computed by means of *XRT*. Comparison of calculated and measured flux densities in Fig. 3 ▸(*a*) indicates that the effective relative energy bandwidth of the beam exiting the monochromator through the centered 1 mm × 1 mm aperture is very close to 0.1%. Although according to *XRT* the relative energy bandwidth increases at high energies, we can use d*E*/*E* = 0.001 for a quick but reliable upper estimate of the photon flux based on the theoretical spectral flux curves. In practice, the peak flux density of almost 8 × 10^10^ photons mm^−2^ s^−1^ is reached at 50 keV with the wiggler field 3.3 T and two 2.2 mm-thick SiC attenuators.

## 3. Endstations and detector pool

### 3.1. BMIT-BM endstations

**Figure 4:**
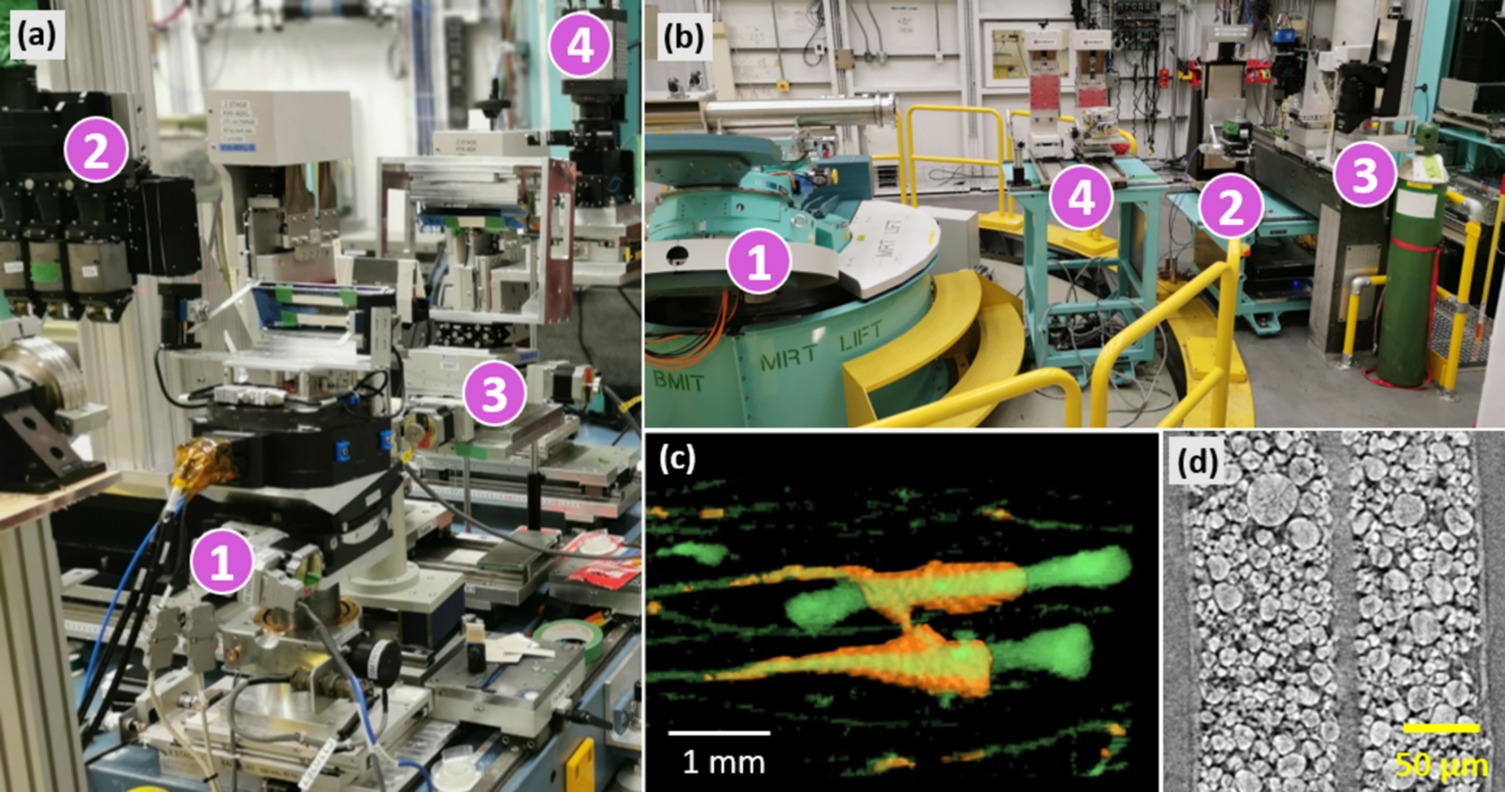
Experimental stations and examples of results. Panel (*a*) shows BMIT-BM during studies of Laue diffraction in bent Si crystals. The main sample positioner, label (1), is repurposed to support the upstream crystal, the white-beam microscope (2) is moved out of the beam, and downstream mounting points are used to support the second crystal and the monochromatic beam microscope (labeled as 3 and 4, respectively). Panel (*b*) shows the interior of the imaging hutch on BMIT-ID beamline. The heavy-duty LAPS/MRT sample positioning system is labeled (1), the primary sample positioning system marked as (2). The microscope, macroscope, label (3), and the large-field-of-view detector can be seen on the right side of the image. Label (4) points to the auxiliary positioning systems. Panels (*c*) and (*d*) contain examples of results from BM and ID beamlines, respectively (see the text in Section 5 for details).

The upstream optical table at 20 m carries an ionization chamber, fast imaging shutter, and has space for one positioning system. The main optical table [Fig. 4 ▸(*a*)] at 25 m has four platforms which can be used to support positioning systems. Two upstream platforms are used in all microtomography experiments for sample positioning and white beam microscope support. The sample positioning system features a 90 mm vertical travel range, a goniometer for laminography experiments, Aerotech (USA) ABRS200MP rotary stage with maximum rotation velocity of 360° s^−1^, a weight capacity of 30 kg, and a motorized *XY* stage for fine positioning of the sample within the detector’s field of view. If the *XY* stage is detached, the distance from the computed tomography (CT) stage surface to the X-ray beam is 140 mm which somewhat limits the dimensions of the samples. A more severe constraint on geometry and dimensions of non-trivial sample holders and *in situ* cells stems from the relatively large horizontal source size. For instance, in order to avoid penumbral blurring, the sample-to-detector distance must not exceed 20 cm when working with the pixel size of 2 µm.

On the two downstream platforms several different configurations of positioning systems can be erected for imaging with medium spatial resolution (tens of micrometres) as well as more complex samples and non-standard experiments (such as live animals or compression/tension load frames for *in situ* testing of metal alloys). Directly downstream of the table, an analyzer crystal is mounted on a granite gantry. The diffracted beam can be imaged by means of a positioning system capable of changing both elevation and inclination of the detector.

The total usable length of the main optical table is about 120 cm. There is ample space on both sides on the table for placement of ancillary equipment such as an anesthesia machine, battery cycler, hot air blower, *etc*.

### 3.2. BMIT-ID endstations

There are multiple points inside the BMIT-ID experimental hutch which can be used to mount and position equipment and samples of various dimensions and weight. The interior is dominated by the large animal positioning system [LAPS, label 1 in Fig. 4 ▸(*b*)]. It is located at 56 m away from source, has positioning accuracy of ±5 µm, vertical travel range of 700 mm, and can rotate samples with velocity of up to 360° s^−1^. For the purposes of microbeam irradiation research, a Kappa geometry goniometer (Huber Diffraktionstechnik GmbH, Germany) was mounted on top of the LAPS. The resulting useful payload capacity is therefore limited to 120 kg. Additionally, because of the bulky counterweight of the Kappa goniometer the sample-to-detector distance cannot be set smaller than 1.5 m. The resulting penumbral blurring limits the system’s imaging capability so that only voxel sizes larger than 30 µm can be reasonably applied. Just recently we have used this system to make a tomography scan of more than a meter long fossil with voxel size of 42 µm.

As the majority of researchers coming to BMIT are interested in spatial scales of 10 µm and smaller, the optical table located at 59 m from the source is used in everyday operation [label 2 in Fig. 4 ▸(*b*)]. A new sample positioning system features submicrometre positioning accuracy, 250 mm of vertical and horizontal travel range, supports laminography geometry, and can accept payloads of up to 20 kg. Commonly used equipment and gas bottles can be positioned sufficiently close to the sample around the main table. Downstream from the positioning system, a microscope, macroscope and a large-field-of-view detector are located in sequential positions [label 3 in Fig. 4 ▸(*b*)]. The default positions are such that penumbral blurring is smaller than the respective pixel size of each detector. The distance between sample and detector can be changed in the range of 5 mm to 2 m, although certain combinations and ranges may require manual reassembly of the support structure and subsequent realignment. As the microscope is equipped with an X-ray-transparent glassy carbon mirror, in certain scenarios simultaneous acquisition of data at two different spatial scales is possible.

Two additional mounting points for ancillary equipment such as the ionization chamber or collimator are available [label 4 in Fig. 4 ▸(*b*)]. In total, up to four items can be positioned in line on the motorized stages and aligned with all commonly used degrees of freedom.

SOE1 is equipped with two overhead cranes with 750 kg load capacity each. The door frame is 3 m × 2 m and there is direct access to the hutch via the corridor/loading bay in the LAB2 lab so that bulky equipment can be handled if necessary.

### 3.3. Detector pool

**Figure 5:**
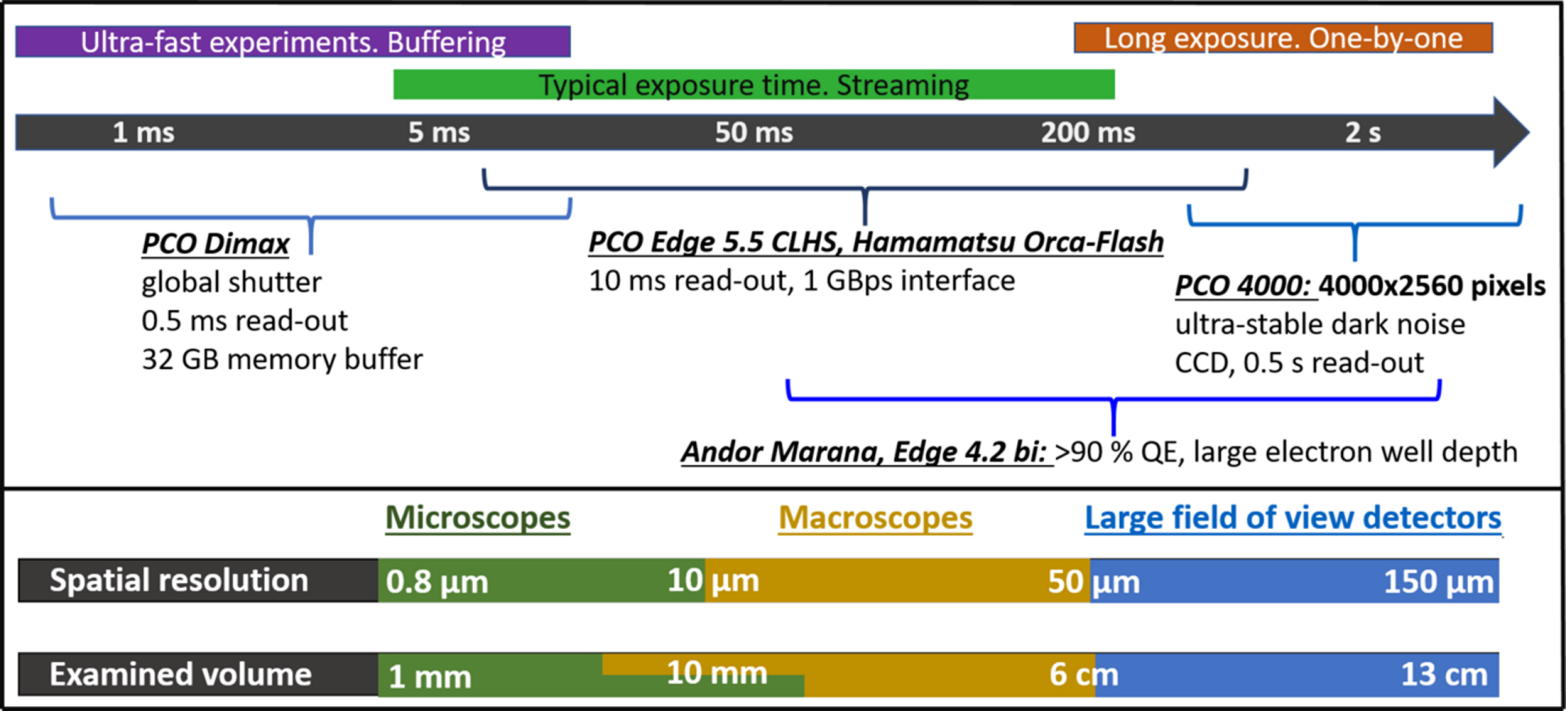
Available instruments for imaging at various spatial and temporal scales. Spatial resolution is derived from 2D images of line-space pairs of suitable resolution test targets. The diameter of the reconstruction circle (*e.g.* horizontal extent of the examined volume) can be doubled by means of offsetting the tomographic axis of rotation from the center to the edge of the detector and making a full 360° rotation (a technique also known as the half-acquisition mode). The vertical extent of the volume examined in one scan is limited by either beam height or detector field of view. An up-to-date list of various detector configurations is maintained at https://bmit.lightsource.ca/tech-info/detectors/.

With one exception all imaging detectors used in experiments are of an indirect type (Koch *et al.*, 1998 ▸). Sample dimensions vary from hundreds of micrometres to tens of centimetres and acquisition time can be anywhere between a fraction of a second to many hours. BMIT offers a number of optical systems and cameras which can be combined to achieve the desired spatial resolution and acquisition speed (Fig. 5 ▸).

The microscopes are standard white-beam-compatible instruments (Douissard *et al.*, 2012 ▸) from Optique Peter (Lentilly, France). The microscope on the BMIT-BM beamline is equipped with three objective heads to enable rapid change of magnification. On the BMIT-ID beamline, a single-objective instrument from Optique Peter is used. It can take up to 30 min to change magnification and realign the scintillator and axis of rotation. Objectives with the magnification 2×, 5×, 7.5×, 10×, and 20× are available and all used on the BM beamline; on the ID beamline, however, the exposure time required to achieve good signal to noise ratio with 10× and 20× objectives is impractically long. Each objective is normally used with a single-crystal scintillator of a matching thickness so that line-space pairs with a 4-pixels-large period can be resolved in images of the 2D JIMA spatial resolution target. Thin scintillators (6–20 µm) are epitaxially grown LSO:Tb crystals from ESRF (Martin *et al.*, 2009 ▸); 50 µm and thicker scintillators are LuAG:Ce crystals from Crytur (Turnov, Czech Republic).

The macroscopes are AA40 and AA60 tandem systems from Hamamatsu Photonics (Japan). The objective and imaging lenses with 50, 80, and 100 mm focal distances are used interchangeably to achieve demagnification of 0.5× or magnifications of ∼1×, and 2×. In-house modifications include a tube lens attachment that is compatible with F-mount cameras from PCO as well as tiltable scintillator holders for the AA40 system (the lack of which in the original Hamamatsu design yields very poor image quality with fully opened apertures). While the light collection efficiency of photolenses is an order of magnitude larger than that of a low-resolution microscope objective, there are two limitations of AA systems which are difficult to overcome: (1) very strong vignetting, even with closed apertures and (2) very low values of the modulation transfer function for spatial frequencies of 30 line-space pairs per mm and above. Nevertheless, thanks to a sufficiently large field of view and high light collecting efficiency, the AA macroscopes are still commonly used for biomedical studies involving imaging of small-to-medium size model organisms like mice and rabbits (and even small piglets).

Lastly, since BMIT’s beams in experimental hutches are wide enough to illuminate up to 15 cm of a sample horizontally, large-field-of-view detectors are deployed on both beamlines. On BMIT-BM, a ten-year-old system from Photonic Science (United Kingdom) is still being used, which comprises a CMOS sensor (4000 × 2000 pixels, pixel size of 25 µm) coupled to a Gadox scintillator screen using a fiber-optic taper. This detector produces images of high quality; it has received a substantial radiation dose over the years but it is still working satisfactorily. For imaging of large objects on the BMIT-ID beamline, an indirect detector was designed by Optique Peter. A unique capability of the system is the continuous zoom in the 0.24 to 0.15× demagnification range, which is extremely convenient in practice. On the downside, the highest effective numerical aperture of the lens is only 0.03. On the upside, this rather low numerical aperture results in a very large depth of focus, and, as a consequence, LuAG:Ce scintillators with 1 mm thickness can be easily used with no impact on the spatial resolution. Other scintillators used are Gadox screens: a 40 µm-thick scintillator custom made by Photonic Science, as well as DRZ-HR and DRZ-fine screens from Mitsubishi Chemical (Japan). Despite the low numerical aperture, the signal-to-noise ratio and overall quality of µCT images of swine chest and lungs obtained with the DRZ-fine screen is comparable with data obtained using the industrial Dalsa Shad-o-Box 3K HS flat panel under the same conditions and same total exposure time. The Optique Peter zoom system can accept scintillators with up to 150 mm × 80 mm area, which was once used to record 3D X-ray diffraction patterns (Poulsen, 2012 ▸) of a Zr alloy.

There are a number of imaging sensors which can be coupled to microscopes and macroscopes. The most frequently used device in the past four years has been a PCO Edge 5.5 CLHS camera. Despite being very old, the PCO4000 CCD is also frequently requested thanks to its large sensor and comparatively rapid read-out. The PCO Dimax HS4 is essential for studies of fast processes and it is an excellent all-round sensor in general. Lastly, cameras with the highest quantum efficiency, namely Andor Marana 4.2B-11 and PCO Edge 4.2 bi, were procured for dose-sensitive applications such as imaging of live animals. They are also used in cases when the detection efficiency is prioritized over the field of view and good dynamic range is required.

## 4. Beamline infrastructure

### 4.1. Computing resources

A new data acquisition and reconstruction system has been recently deployed which substantially improved the efficiency and accessibility. At a high level, there are two applications – one for data acquisition and one for data reconstruction. At the lower levels, the system is modular with critical libraries written in C and fast synchronization enabled by programmable CT stage controllers. The new system is also completely distributed with various roles assigned to dedicated servers connected by a high-performance network.

The new data acquisition software stack comprises the following major components:

(i) The frontend is a *PyQt5*-based graphical user interface which allows users to perform all commonly encountered types of CT scans.

(ii) In the back-end, *Concert* (https://github.com/ufo-kit/concert; Vogelgesang *et al.*, 2016 ▸) controls the data acquisition flow.

(iii) Communication with cameras is handled by means of *libuca* (Unified Camera Access, https://github.com/ufo-kit/libuca).

(iv) Communication with motors happens via an EDC (EPICS Devices for Concert) layer.

A National Instruments/LabView-based system is also available, which can be convenient in cases when researchers need to synchronize their equipment with the beamline hardware. One example is a respiratory-cardio monitor, required for *in vivo* imaging, which must be synchronized with the CT stage, camera, and fast imaging shutter. It is worth noting that, when LabView is used, synchronization with cameras happens by means of external triggers only.

Data reconstruction, as well as image processing and suppression of artifacts, is carried out using the *tofu* software kit (Faragó *et al.*, 2022 ▸). A dedicated reconstruction server is reserved for each beamline for quality assurance and quick preview of the results during experiment. This is crucial for *in vivo* and *operando* studies. Additionally, two high-performance workstations have been deployed for post-experiment reconstruction, segmentation, visualization, and analysis of results. Servers are located in a separate data analysis room. Users can connect to them remotely as well using NoMachine, and retrieve processed data with the help of the Globus network service. This mode of operation became extremely popular during the COVID-19 pandemic, and has remained a preferred mode of access ever since.

Compared with the old data acquisition system, training of users has become a lot simpler thanks to a single application for data acquisition and another single application for data reconstruction. From a developer’s point of view, it is easy to connect the data acquisition and reconstruction operations and thereby improve automation, since *Concert*, *libuca*, *ufo*, and *tofu* are all parts of the wider *ufo-kit* framework. For instance, on-the-fly preview of the CT slices is possible (a demonstration video can be found in the supporting information). Interestingly, this functionality is not very popular among our user community. Normally researchers need to preview data with phase-retrieval applied and ring artifacts removed. Depending on the data size, these operations used to take a couple of minutes to tens of minutes on beamline servers equipped with ordinary gaming GPUs. With the recent addition of a GPU node to the CLS computing cluster, reconstruction time has been significantly reduced.

### 4.2. Support laboratories and equipment

There is a dedicated laboratory adjacent to each of the beamlines, each of which is equipped with basic equipment and consumables including an automatic injection pump, two anesthesia machines, and an animal holding areas for mice, rats, piglets, and rabbits. The lab associated with the BMIT-BM beamline is risk group level 2 and can be used to handle biohazardous materials using a biosafety cabinet. Animals can stay in the labs for short periods of time, and long-duration stays can be arranged with the University of Saskatchewan upon request. There is a Biovet system for gated imaging based on respiration and heartbeat. A PTW dosimeter (PTW Freiburg GmbH) and calibrated pinpoint ion chambers are available to monitor the dose. In addition to the BMIT laboratories, CLS has several shared laboratories that users can book in advance. Some available equipment includes fluorescence microscopes, optical microscopes, dissecting microscopes, a cryostat microtome, paraffin microtome, atmospheric and CO2 incubators, freeze dryer, drying oven, a live plant growth chamber, two hydrogen generators. The ‘energy materials *in situ* hub’ contains some instruments (such as argon glove boxes, potentiostats, and battery cyclers) that can be used for dynamic experiments. A complete list of available equipment can be found on the CLS website (https://www.lightsource.ca/facilities/laboratories-equipment.php). Lastly, a ThermoFisher Quattro 5 environmental scanning electron microscope with two Bruker XFlash detectors for energy-dispersive spectroscopy is also available at CLS.

## 5. Experimental program and highlights

Instruments and techniques available at BMIT have been successfully applied to numerous biomedical and preclinical studies using both excised tissue specimens and animal models ranging in size from zebra fish and mice to piglets. BMIT’s powerful imaging capabilities are also routinely utilized by experts for research of alloys and composite materials, renewable energy, environmental science, and many other applications.

### 5.1. Biomedical research

Several live-animal models are imaged routinely at BMIT, including mice, rats, rabbits, piglets, and, in the past, small dogs. The diseases studied include osteoporosis, osteoarthritis, cystic fibrosis, prostate cancer, *etc*. Cooper and co-workers recently imaged live rabbits using a safe radiation dose, followed by recovery and second-time-point imaging *ex-vivo* to measure the progression of basic multicellular units in cortical bone over time (Loundagin *et al.*, 2024 ▸). Volumetric rendering shown in Fig. 4 ▸(*c*) depicts evolution of the osteoporosis in the tibia bone. The orange and green colors show the same region imaged two weeks apart. Data were acquired with 37 keV monochromatic beam, 200 µm-thick LuAG:Ce scintillator, 0.5× demagnification, and Hamamatsu ORCA-Flash4.0 V2 sensor; total exposure time is 30 s with an extra 40 mm of plexiglass inserted in the beam in order to satisfy the dose constraints.

Another example of live-animal research is cystic fibrosis in the piglet animal model. Various trials for a better understanding of the disease as well as finding a potential treatment are underway (Luan *et al.*, 2017 ▸, 2019 ▸). In addition to live-animal research, many other studies performed at BMIT have yielded valuable results. Ladak and colleagues studied the anatomy and neurosensory structures of the human inner and middle ear using multiscale resolution for the ultimate goal of designing more efficient hearing implants (Li *et al.*, 2021 ▸, 2022 ▸; Mei *et al.*, 2020 ▸). One of the advantages of partially coherent beam at the synchrotron is the ability to image soft tissues that are almost transparent to the hard X-rays. Imaging soft hydrogel scaffolds used in tissue regeneration research (*e.g.* for nerve regeneration) is an example of the research that benefits from X-ray phase contrast (Ning *et al.*, 2021 ▸).

### 5.2. Material science

Hard X-rays are ideal for imaging of radiopaque materials relying on the attenuation of X-rays, as well as for imaging soft materials that benefit mainly from phase contrast. An example of soft materials research is the microstructural characterization of polyurethane foams to improve their mechanical response following impact (Bhagavathula *et al.*, 2022 ▸). BMIT-BM was used to characterize the microstructure of macroporous graphene oxide scaffolds that have various applications in medicine (Chen *et al.*, 2022*b* ▸) or in fuel cells (Capilli *et al.*, 2022 ▸). An example of a radiopaque sample includes AlSi10Mg alloy (imaged on BMIT-ID) to characterize the fracture behavior of an alloy that was made by laser powder bed fusion (Chen *et al.*, 2022*a* ▸). Efforts are underway at the BMIT beamlines to add capabilities for enabling complex *in situ* experiments. Acceptance tests of a CT-compatible load frame took place in late 2023.

### 5.3. Batteries and fuel cells

*In situ* and *operando* imaging is vital for characterizing microstructural changes in battery materials. Bond *et al.* studied the effects of charge-discharge cycling in lithium-ion pouch cells by using *in situ*, multiscale µCT to investigate electrolyte depletion and electrode material degradation due to long-term cycling (Bond *et al.*, 2022 ▸). In a fragment of the reconstructed CT slice shown in Fig. 4 ▸(*d*) the microcracks and pores in cathode particles are clearly visible after multiple charge–discharge cycles. This data set was acquired with white beam filtered by 0.8 mm Al and 0.25 mm Cu, 6 µm-thick LSO:Tb scintillator, 18× magnification, PCO Edge 5.5 CLHS sensor; voxel size 0.34 µm × 0.34 µm × 0.34 µm and takes 20 min to acquire one CT set with such settings. Other researchers utilize high-resolution µCT for characterizing solid-state batteries, including lithium metal (Yang *et al.*, 2022*b* ▸). Moreover, fuel cell samples are frequently imaged at BMIT, from microstructural characterization of emerging materials (Fu *et al.*, 2021 ▸; Yang *et al.*, 2022*a* ▸) to *operando* imaging of the polymer electrolyte membrane (Guan & Bazylak, 2023 ▸).

### 5.4. Plant biology, agriculture and food chemistry

In the last few years, imaging of plants and agricultural samples has become popular. Most of the samples are scanned at the BMIT-BM beamline with filtered white beam, due to the higher photon flux and lower X-ray energy. Typical agricultural samples include seeds, cannabis, flowers, whole small trees, plant-based meat products, and soil. Recently, high-resolution µCT was used to study the effects of adding minor non-triglyceride lipids to cocoa butter and the resultant changes in micro-structure of the recrystallized chocolate (Chen *et al.*, 2021 ▸). Another project of agricultural relevance is focused on alternative sources of energy that are low in carbon and sourced from agricultural biomass. The researchers prepare pellets from various agricultural biproducts and aim to optimize the pelletization process (Sarker *et al.*, 2022 ▸; Azargohar *et al.*, 2022 ▸).

### 5.5. Environment

Recently, researchers from University of Calgary used BMIT-ID beamline to look at the composition and physical characteristics of rocks from oceanic crust (Pujatti *et al.*, 2023 ▸). They looked at the pore size and pore network within these samples and discovered that they are rich in magnesium (olivine) which, combined with the large pore structure, leads to the release of magnesium that is potentially useful for carbon sequestration. In another study, Leontowich *et al.* used *K*-edge subtraction imaging to look at the fragmentation pattern in lead-based bullets used in hunting (Leontowich *et al.*, 2022 ▸). They discovered that thousands of small micrometre-sized (and perhaps sub-micrometre) bullet fragments were scattered in the sample when lead bullets were used. These fragments are of concern as they can be digested by humans and scavenger animals, and also contaminate soil.

### 5.6. Paleontology

Canada possesses several large collections of valuable paleontological specimens. X-ray µCT can give insights in the anatomy of extinct specimens (Brinkman *et al.*, 2023 ▸) as well as test various evolution hypotheses (Powers *et al.*, 2021 ▸). One of the skulls featured in the latter study is the largest sample scanned at BMIT in its entirety. It has dimensions of 17 cm × 14 cm × 45 cm and homogenization of the absorption protocol (Sanchez *et al.*, 2013 ▸) was utilized to achieve a suitable signal to noise ratio. At the microscopic scale, a large number of amber inclusions have been scanned and some interesting findings have been recently reported (Mitchell *et al.*, 2023 ▸).

### 5.7. Instrumentation

BMIT has contributed to the development of a number of instruments and techniques. Prime examples are the beam-expanding monochromator (Martinson *et al.*, 2017 ▸) and spectral imaging using bent Laue crystals (Zhu *et al.*, 2014 ▸; Bassey *et al.*, 2016 ▸). A real-time electron beam position and angle monitoring system was also developed (Samadi *et al.*, 2019 ▸). High-resolution rocking curve imaging is routinely used to control the quality of diamond crystals which are intended for high-energy particle physics experiments. Recently, an in-house-built radiometer was commissioned on BMIT-ID, which allows for direct measurements of absolute photon flux density and calibration of other devices such as diodes. Lastly, BMIT has also been used in the early stages of development for a novel coherent scatter X-ray imaging system (Dydula *et al.*, 2019 ▸).

### 5.8. Facility access

There are two primary access mechanisms: one for industry clients and one for academic researchers who can obtain access to the instruments for a nominal fee of one dollar per day. Academic users can submit proposals twice a year, normally in February and August. Applications are peer reviewed by an international panel of experts and time is allocated based on the scientific merit. The duration of experiments can vary greatly depending on the type of study. For example, µCT inspection of a several simple samples often can be completed in one CLS shift (8 h), while *in situ* and *in vivo* studies can take up to six to ten days (sometimes spread over the course of several weeks and even months). CLS proposals are valid for four cycles (or two years) which gives users an opportunity to make long-term plans or simply request more time if the initial goal was not fully achieved in the first experiment. Industrial clients of the facility can access proprietary beam time on a fee-for-service basis, where CLS staff often carry out experiments on behalf of clients.

## 6. Discussion and plans for improvement

Despite the great utility of the BMIT beamlines across a number of applications, including those discussed above, there are limitations which compromise the quality of some results or make it impossible to perform certain studies. The fundamental limitations are: the large source size, very low photon flux density in the low-energy monochromatic beam, and the lack of access to high-energy white beam on the BMIT-ID. The source size is defined by the storage ring and it is too large to fully exploit propagation-based phase contrast, particularly at higher X-ray energies. Penumbral blurring also limits the amount of space between samples and detector. While not much can be done about the source size, it is entirely possible to both enable sub-hour, high-resolution CT scans with monochromatic beam on BMIT-BM as well as to increase the flux density on the BMIT-ID beamline. To achieve the former goal, funding for a double multilayer monochromator for the BMIT-BM beamline has been requested. As for the BMIT-ID beamline, numerical simulations are ongoing and a test stand was made in order to determine the optimal thickness, the asymmetry cut, and to study the bending of Si crystals. Pre­liminary results indicate that it is very feasible to increase the flux density fourfold at photon energies of 25–40 keV by reducing the thickness of the first crystal, increasing the asymmetry cut, and ensuring a uniform cylindrical bending. A substantial increase in monochromator efficiency at higher photon energies is practically impossible. One proposed idea is to enable access to the filtered wiggler beam and erect a dedicated imaging station in the POE3 hutch, which has a sufficient radiation shielding. The design study for this upgrade has been completed and funding applications are ongoing. The proposal for this upgrade specifies that flux densities of up to 10^12^ photons mm^−2^ s^−1^ at photon energies above 100 keV can be achieved and that the mean energy of the filtered wiggler beam can be pushed to 200 keV.

In addition to major X-ray optics upgrades, there is ongoing improvement and optimization of existing beamline components such as filters, vacuum windows, and sample positioning stages. Good examples are improved clamping system of high-heat-load filters and new long-travel range, high-precision vertical positioning stage. Slip ring systems are being incorporated into primary positioning systems as well. An upgrade of the detectors is another way to increase the performance of any beamline. A macroscope is under construction by Optique Peter which will combine the quality of microscope objectives with the numerical aperture of photography lenses. Thanks to the superior quality and detection efficiency this instrument will bring functional live imaging of small animals to a new level. In addition to upgrading optical systems we are also purchasing latest-generation sCMOS cameras, which have higher resolution and larger sensors, better quantum efficiency, and faster read-out times compared with old devices.

## 7. Conclusion

BMIT is unique in North America in offering a high-energy, monochromatic X-ray beams with a very large cross-section. It is also one of just a handful of beamlines in the world where experiments with live animals are possible. Here we have reported the key parameters of the BMIT beamlines, the supporting infrastructure, and recent upgrades that have substantially enhanced the beamlines’ µCT capabilities. Improvements have also been made to the detector pool, sample positioning systems, and data acquisition/reconstruction software. These changes have been critical for multi-scale imaging and increased throughput. They have also enabled many new *in situ* and *operando* studies as well as many other experiments which were not possible before. This is reflected in the publication list and continuously broadening community of researchers working in different societal areas.

We have discussed the beamline limitations with the two fundamental issues being rather large source size and a very low flux in the monochromatic beams at energies below 35 keV. The latter problem can be solved by adding a double multilayer monochromator to the BMIT-BM beamline and optimizing a Laue–Laue monochromator on the BMIT-ID beamline. These ideas constitute our primary future upgrade plans.

## Supplementary Material

Data acquisition interface and demonstration of a fast scan and on-the-fly tomographic reconstruction. DOI: 10.1107/S1600577524005241/ye5043sup1.avi

## Figures and Tables

**Table 1:** Key parameters of the BMIT-BM beamline

Source parameters	
Magnetic field	1.35 T
Critical energy	7.6 keV
Effective source size (H × V), FWHM	400 µm × 150 µm (Samadi *et al.*, 2022 ▸)

Beam parameters, 25 m from the source	
Horizontal size (flat-top intensity profile)	20 cm
Photon flux density in monochromatic beam @ 20 keV	1.5 × 10^9^ photons mm^−2^ s^−1^
Vertical size of the 20 keV monochromatic beam	4.0 mm @ FWHM
Photon flux density in the white beam: Al 0.8 mm filter, mean energy 20 keV	∼9 × 10^12^ photons mm^−2^ s^−1^
Vertical size of the white beam	3.2 mm @ FWHM

**Table 2:** Key parameters of the BMIT-ID source computed in *SPECTRA10* (the source size was verified by observations of penumbral blurring)

Source parameters		
Magnetic field	2.5 T	3.7 T
Critical energy	14 keV	21 keV
Source size (H × V), FWHM	0.8 mm × 0.1 mm	1.2 mm × 0.1 mm

Beam parameters, 59 m from source	@ 30 keV	@ 100 keV
Beam size (H × V), FWHM	160 × 10 mm	180 × 7 mm
Photon flux [photons mm^−2^ s^−1^ (0.1% bandwidth)^−1^]	3 × 10^11^	7.5 × 10^10^
